# Acute aerobic exercise: an intervention for the selective visual attention and reading comprehension of low-income adolescents

**DOI:** 10.3389/fpsyg.2014.00575

**Published:** 2014-06-11

**Authors:** Michele Tine

**Affiliations:** Poverty and Learning Lab, Education Department, Dartmouth CollegeHanover, NH, USA

**Keywords:** interventions, poverty, reading comprehension, selective attention, adolescent

## Abstract

There is a need for feasible and research-based interventions that target the cognitive performance and academic achievement of low-income adolescents. In response, this study utilized a randomized experimental design and assessed the selective visual attention (SVA) and reading comprehension abilities of low-income adolescents and, for comparison purposes, high-income adolescents after they engaged in 12-min of aerobic exercise. The results suggest that 12-min of aerobic exercise improved the SVA of low- and high-income adolescents and that the benefit lasted for 45-min for both groups. The SVA improvement among the low-income adolescents was particularly large. In fact, the SVA improvement among the low-income adolescents was substantial enough to eliminate a pre-existing income gap in SVA. The mean reading comprehension score of low-income adolescents who engaged in 12-min of aerobic exercise was higher than the mean reading comprehension score of low-income adolescents in the control group. However, there was no difference between the mean reading comprehension scores of the high-income adolescents who did and did not engage in 12-min of aerobic exercise. Based on the results, schools serving low-income adolescents should consider implementing brief sessions of aerobic exercise during the school day.

## INTRODUCTION

The disparity in educational attainment between low- and high-income adolescents has widened by more than forty percent in the last 50 years (Reardon, 2013). Low-income adolescents fall behind their high-income peers on standardized tests across a variety of academic domains and on other educational attainment measures, including but not limited to grade point average, college enrollment, and college completion rates (Bailey and Dynarski, 2011; National Assessment of Educational Progress, 2014). In response, cognitive research has begun to focus on identifying cognitive processes that may underlie the income-achievement gap. For example, it has been determined that low-income students exhibit a variety of attention deficits compared to high-income students (e.g., Mezzacappa, 2004; Farah et al., 2005, 2006; Noble et al., 2005, 2007). Selective visual attention (SVA) is one specific attention deficit that has been identified among low-income students; it is the ability to remain focused on a relevant visual input while suppressing other irrelevant inputs (Mezzacappa, 2004; Hackman and Farah, 2009). Not surprisingly, SVA is critical for learning in any academic domain (Kruschke, 2005; Duncan et al., 2007; Rueda et al., 2010; Stevens and Bavelier, 2012). If a student cannot pay attention to information and ignore distractions during class, they will not be able to process the information and eventually retain it (Baddeley and Hitch, 1994; Kruschke, 2005).

Considering the role SVA plays in learning, we recently tested an intervention designed to improve the SVA of low-income 10 year olds (Tine and Butler, 2012). We tested if low-income children’s SVA would improve after engaging in an acute session of aerobic exercise, a single session of exercise that lasts <20 min and is completed at a cardiovascular intensity level of 70–80% of an individual’s maximum heart rate (American Heart Association, 2009). The SVA of low-income children did improve significantly after the acute session of aerobic exercise. In fact, the improvement seen in low-income children was even greater than the improvement seen in high-income children (Tine and Butler, 2012). The results of the study served as an impetus for primary schools serving low-income children to implement sessions of acute sessions of aerobic exercise during the school day (Tine and Butler, 2012).

It is now important to determine if an acute session of aerobic exercise is also an effective intervention for improving the SVA of low-income *adolescents*. There are multiple reasons that make it important to test the impact of an acute bout of aerobic exercise on adolescents, instead of simply assuming its effects on low-income children generalize to an older population. First, many cognitive models explicitly highlight how the impact an event has on an individual is dependent on the individual’s cognitive developmental stage (e.g., Casey et al., 2008; Bialystock and Craik, 2010). Adolescents and children are at distinct cognitive developmental stages of SVA; adolescents have fully developed SVA, but children’s SVA is still developing (Plude et al., 1994; Ridderinkhof and van der Stelt, 2000). It is possible, then, that an acute bout of aerobic exercise might affect individuals with fully developed SVA (i.e., adolescents) differently than it affects individuals with still developing SVA (i.e., children). A second reason acute aerobic exercise might affect low-income adolescents differently than low-income children is physiological. The chronic stress of poverty deregulates the physiological systems that release cortisol (Kunz-Ebrecht et al., 2004; Cohen et al., 2006; Miller et al., 2007; Chen et al., 2010). Low-income adolescents experience more chronic stress in their lifetime than low-income children (Evans and English, 2002; Evans and Schamberg, 2009). In turn, it seems possible that higher levels of chronic stress could lead low-income adolescents to have more severely deregulated physiological systems than low-income children. This is relevant because acute aerobic exercise typically causes a release of cortisol (Viru and Viru, 2004; Ferris et al., 2007; Griffin et al., 2011). Taken together, low-income adolescents with more deregulated physiological systems might have a different physiological response to acute aerobic exercise than low-income children with less deregulated physiological systems. Measuring physiological responses was beyond the scope of this paper, but the potential role of chronic stress underscores how important it is to determine how acute aerobic exercise impacts low-income adolescents at a behavioral level, instead of inappropriately generalizing results drawn from other populations. Therefore, the first goal of the current study was to establish if and how an acute session of aerobic exercise impacts the SVA abilities of low-income adolescents and, for comparison reasons, high-income adolescents.

It is important to acknowledge that interventions other than an acute session of aerobic exercise have been found to effectively improve the SVA of adolescents, but none have been conducted specifically with low-income adolescents. Moreover, the other interventions were particularly time consuming. For example, playing fast-paced action sports improved the SVA of middle- and high-income athletes; the more years an athlete played these sports, the larger the SVA gain they experienced (Nougier et al., 1996; Houmourtzoglou et al., 1998; Lum et al., 2002). However, a minimum of a year of participation in a fast-paced action sport was necessary to see an improvement. Playing action-based video games also improved middle-income adolescents’ SVA. When they played video games that required them to react to sudden appearances of stimuli among distractors, their performance on separate SVA tasks improved (e.g., Green and Bavelier, 2003; Feng et al., 2007; Hubert-Wallander et al., 2010). Yet, participants had to play the action-based video games every day for weeks at a time to experience the benefits. Participating in sessions of meditation improved SVA of middle-income adolescents, as well, but again participation had to be regular, frequent, and last for months (Jha et al., 2007; Slagter et al., 2007). While the success of these interventions underscores the malleability of SVA, they were time intensive. Time intensive interventions are not practical for practitioners to implement in low-income communities, as they are often limited by severe time and resource constraints. An acute session of aerobic exercise is ideal in that it requires only one session and the session is brief and low-cost.

Yet, the utility of an acute session of aerobic exercise as an intervention is contingent on the benefit lasting beyond the brief exercise session itself. Therefore, the second goal of the current study was to determine if the potential SVA benefits caused by acute aerobic exercise last 45-min after the exercise session ends. It was hypothesized that the SVA benefits would last 45-min for both high- and low-income participants. This hypothesis was based on previous research conducted with high-income participants that found that acute aerobic exercise improved cognitive control until the participants’ heart rates returned to within 10% of baseline, which took on average 48-min (Hillman et al., 2003). Cognitive control and SVA are both domain general executive functions and are positively correlated, making it seem likely that if an improvement in cognitive control lasted approximately 48-min after acute aerobic exercise, an improvement in SVA might also last for a similar duration (e.g., Yantis, 2008).

An acute session of aerobic exercise has even more utility as an intervention if it is also associated with better performance on academic tasks. None of the aforementioned exercise interventions measured academic performance. Therefore, the third goal of the current study was to determine if low- and high-income adolescents who engaged in acute aerobic exercise would have better reading comprehension than those who did not exercise. Reading comprehension was specifically selected as the academic outcome measure for two reasons. First, there is a pressing need to identify reading comprehension interventions of low-income adolescents, as the performance gap between low- and high-income adolescents’ reading comprehension is the largest it has been in 25 years (Reardon, 2013). Second, SVA and reading comprehension are highly correlated, making it seem likely that SVA benefits from acute aerobic exercise, reading comprehension might also benefit (Asbjornson and Bryden, 1998; Casco et al., 1998; Klein and D’Entremont, 1999; Sperling et al., 2005; Ziegler et al., 2005; Franceschini et al., 2012). Therefore, it was hypothesized that the reading comprehension scores of low- and high-income adolescents who engaged in an acute session of aerobic exercise would be higher than the reading comprehension scores of low- and high-income adolescents in the control condition.

In summary, the current study aimed to test an acute session of aerobic exercise as an effective, lasting intervention for low- and high-income adolescents. Based on prior research, it was hypothesized that (1) an acute session of aerobic exercise would improve the SVA of high- and especially low-income adolescents, (2) the SVA improvement would last at least 45-min for adolescents of both income levels, and (3) the reading comprehension scores of low- and high-income adolescents who engaged in an acute session of aerobic exercise would be higher than the reading comprehension scores of low- and high-income adolescents in the control condition.

## MATERIALS AND METHODS

The materials and methods were approved by and conformed to the regulatory standards of the Committee for the Protection of Human Subjects, which is the Institutional Review Board of the institution where the data was collected.

### PARTICIPANTS

Participants (*n* = 85) included adolescents enrolled in a highly selective liberal arts college (age range: 17 years, 4 months – 21 years, 6 months) who responded to recruitment posters placed throughout campus. The experimental condition (*n* = 46; 26 female, 20 male) was comprised of 22 low-income participants and 24 high-income participants. The control condition (*n* = 39; 20 female, 19 male) was comprised of 21 low-income participants and 18 high-income participants. Participants were considered low-income if they grew up in a residence for the majority of their childhood that had an annual household income that fell below 133% of the federal poverty line (or <$31,720.50). They were considered high-income if grew up in a residence for the majority of their childhood that had an annual household income at least 175% above the federal poverty line (or more than $41,737.50). These figures reflect the income eligibility guidelines set by U.S. Department of Agriculture for social services (U.S. Department of Agriculture, 2014). Six additional participants were excluded from analyses because they did not indicate their family income level, did not spend the majority of their childhood in a household of one consistent income level, or indicated that their family income level fell above the low-income threshold of $31,720.50 but below the high-income threshold of $41,737.50. Participants with learning disabilities were excluded from participating in the study.

### MEASURES

#### Physical characteristics

***Age, height, weight.*** The age (months), weight (lbs.), and height (in.) of each participant was self reported.

***Body mass index (BMI).*** Body mass index (BMI) was calculated with the formula: weight (lbs.)/[height (in.)]^2^ × 703, as suggested by the Center for Disease Control and Prevention (2013).

***Resting heart rate.*** Participants sat still in a crossed-legged position with their eyes closed for 5-min. Then, their resting heart rate was measured with an Impact Sports Epulse Strapless Heart Rate Monitor.

***Maximum heart rate.*** Maximum heart rate was calculated with the following formula: HR max = 208 - (0.7*age), as suggested by Tanaka et al. (2001).

***Target heart rate range.*** According to the American Heart Association (2009), exercise is defined as aerobic when a person falls within 70–80% of their maximum heart rate (American Heart Association, 2009). Therefore, the target heart range for each participant was calculated using the following formula: (HR max*0.7) -to- (HR max*0.8).

#### Selective visual attention

The d2 Test of Attention was used as the SVA pretest, immediate posttest, and delayed posttest. The d2 Test of Attention is a one-page paper and pencil cancelation task of 14 rows (trials), each with 47 interspersed “p” and “d” characters (Brickenkamp and Zillmer, 1998). The characters have one to four dashes that are configured individually or in pairs above and/or below each letter. The target character is a “d” with two dashes (hence “d2”), regardless of whether the dashes appear both above the “d,” both below the “d,” or one above and one below the “d.” Thus, a “p” with one or two dashes and a “d” with more or less than two dashes are distracter characters. The task was to cross out as many target characters as possible, moving from left to right, with a time limit of 20 s per trial. No pauses were allowed between trials. The internal test-retest reliability of the d2 test has been shown to be very high for all parameters (0.95–0.98) and the criterion, construct, and predictive validity have been documented and shown to be strong and stable (Brickenkamp and Zillmer, 1998; Bates and Lemay, 2004). The test was scored using the following procedures, as suggested by the d2 Administration Manual (Brickenkamp and Zillmer, 1998). The total number of characters processed was calculated by summing the total number of characters that fell before the final (either correctly or incorrectly) crossed out character in each trial. Errors of omission were the total number of target characters (i.e., a “d” with two dashes) that were processed, but not crossed out across all 14 trials. Errors of commission were the total number of distractor characters (i.e., any character other than a “d” with two dashes) that were processed and incorrectly crossed out across all 14 trials. The total number of errors was calculated by summing the errors of omission and commission. Finally, the overall SVA score was calculated by subtracting the total number of errors from the total number of characters processed.

#### Reading comprehension task

The reading comprehension task was composed of three reading comprehension passages from Barron’s GRE 18th Edition Test Preparation book (Green and Wolf, 2009). Each passage was approximately 1500 words and was interspersed with 20 multiple choice reading comprehension questions. The passages were administered in multiple sections, such that one section was presented for a participant to read and then taken away before the reading comprehension questions related to that section were administered. This prevented a participant from being able to reread the text while answering the reading comprehension questions. Participants were given as much time as needed to complete the reading comprehension task. No participant took less than 14-min or more than 20-min to complete the task. The reading comprehension score was the percent of items correctly answered. Blank answers were considered incorrect.

#### Stress inventory survey

It was important to obtain information on the number and type of stressors in participants’ lives, as research has shown that it is the chronic stress of poverty that impacts cognitive processes (Evans and Schamberg, 2009). Therefore, an adaptation of the Stressful Life Events Schedule Survey (Williamson et al., 2003) was administered to all participants. On the survey, participants indicated if eighty different stressful life events had happened to them over the course of their life and, for each event that had, how much it affected them on a 4-point Likert scale ranging from “not at all” to “a lot.” Example items include: “My parent was fired from his/her job.”, “I was a victim of a crime.”, “A close friend or family member had health problems.”, “My parents divorced or separated.”, and “I had problems being liked by classmates.” A total life stress score was computed for each participant by summing the Likert scale rating for each event they indicated had happened to them.

#### Participant information survey

The Participant Information Survey asked for self-reported data on each participant’s sex, race, ethnicity, and household income of the residence they grew up in for the majority of their childhood. For household income level, participants selected an income range that best reflected the household income level of the residence they grew up in for the majority of the childhood from nine income range choices. The nine income range choices reflected the nine income ranges used as eligibility guidelines set by U.S. Department of Agriculture for social services (U.S. Department of Agriculture, 2012). This survey was administered after all the other data was collected to avoid stereotype threat effects on the SVA measures, as stereotype threat effects have been shown to affect cognitive processing among young adults when primed about their gender, race, and economic circumstance (e.g., Steele and Aronson, 1995; Spencer and Castano, 2007; Good et al., 2008, respectively).

### PROCEDURE

Before arrival, participants were randomly assigned to the control or experimental condition. A member of the research team rolled a die. If the die landed on an even number the participant was assigned to the experimental condition. If the die landed on an odd number the participant was assigned to the control condition. Upon arrival, the research team obtained informed consent from each participant. Each participant was then fitted with heart rate monitor that they wore throughout the study. After the fitting, participants sat still for 5-min and then the research team recorded the resting heart rate indicated on each participants heart rate monitor. Next, participants self-reported their height, weight, and age. Based on these self-reported measures, a member of the research team calculated each participant’s maximum and target heart rate range using the formulas listed in the Measures section below. Meanwhile, participants completed the SVA pretest. The next 12-min varied for participants in experimental versus control condition.

#### Experimental condition

After participants in the experimental condition completed the SVA pretest, the researchers informed them each of them of their individual target heart rate range. Then, the participants jogged in place for 12-min at a speed that maintained a heart rate within their individual target heart rate range. All participants were able to remain in their target heart rate range for the 12-min exercise session.

#### Control condition

Instead of participating in a 12-min exercise session, participants in the control condition remained seated and viewed a 12-min educational movie clip about the benefits of aerobic exercise. Presenting both conditions with exercise related content was done in an effort to control for the possibility that simply thinking about aerobic exercise may impact one’s cognitive performance.

#### Experimental and control conditions

One minute after the 12-min exercise session or the 12-min film clip, participants completed the SVA immediate posttest. Then, participants in both conditions completed the reading comprehension task. Upon completion of the reading comprehension task, participants read silently to themselves. They had been asked to bring their own reading material with them to the lab and those who did not do so were provided with issues of *Current Directions in Psychological Science* and *Nature*. They read until 45-min had passed from the time they had completed the SVA immediate posttest. The primary purpose of the 45-min delay was to determine if a potential SVA benefit lasted at least 45-min. It also served the purpose of determining if any benefit lasted once one’s heart rate has returned to its resting heart rate (as recommended by Hillman et al., 2003 and Hillman et al., 2009). When 45-min had passed, participants were asked to check their heart rate to ensure that their heart rates were all within 10 beats per minute of their previously measured resting heart rate. Participants then completed the SVA delayed posttest. Finally, participants completed the Stress Inventory Survey and Participant Information Survey. They were thanked and compensated for their time.

## RESULTS

### PARTICIPANT CHARACTERISTICS

Results of ANOVAs showed no statistically significant differences between income levels as a function of resting heart rate, [*F*(1,83) = 0.244, *p* = 0.623], BMI [*F*(1,83) = 0.039, *p* = 0.843], age [*F*(1,83) = 1.697, *p* = 0.196], or time taken to complete the reading comprehension task [*F*(1,83) = 2.468, *p* = 0.120]. There were also no statistically significant differences between the two conditions as a function of resting heart rate, [*F*(1,83) = 1.442, *p* = 0.223], BMI [*F*(1,83) = 0.479, *p* = 0.491], age [*F*(1,83) = 7.343, *p* = 0.108], or time taken to complete the reading comprehension task [*F*(1,83) = 0.984, *p* = 0.324]. Chi Square independence tests showed that the two conditions did not differ from each other in the distribution of sex [χ^2^(2, *n* = 85) = 0.071, *p* = 0.790], race [χ^2^(6, *n* = 85) = 3.535, *p* = 0.473], ethnicity [χ^2^(3, *n* = 85) = 0.513, *p* = 0.774], or household income [χ^2^(9, *n* = 85) = 5.186, *p* = 0.738]. The two income levels did not differ from each other in the distribution of sex [χ^2^(2, *n* = 85) = 1.537, *p* = 0.215], or ethnicity [χ^2^(3, *n* = 85) = 4.972, *p* = 0.083], but did differ from each other in the distribution of race [χ^2^(6, *n* = 85) = 24.255, *p* < 0.001].

### GOAL ONE

**FIGURE 1:**
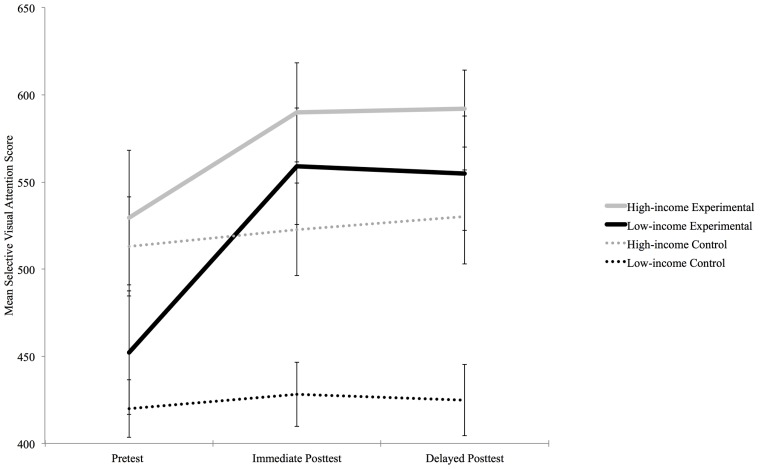
**Selective visual attention pretest, immediate posttest, and delayed posttest scores for high- and low-income participants in the experimental and control conditions**.

A series of analyses were run to determine if and how an acute session of aerobic exercise impacted low- and high-income participants’ SVA. First, a 2 × 2 × 2 mixed-design ANOVA was run with test time (pretest, immediate posttest) as a within-subject variable, and condition (experimental, control) and income (high-income, low-income) as between-subject variables. Results revealed a series of statistically significant effects. First, there were main effects of test time [*F*(1,81) = 121.35, *p* < 0.001, $\eta_{p}^{2}$ine-formula> = 0.60], condition [*F*(1,81) = 25.33, *p* < 0.001, $\eta_{p}^{2}$ = 0.24], and income [*F*(1,81) = 36.44, *p* < 0.001, $\eta_{p}^{2}$ = 0.31]. Second, there were significant two-way interactions between test time and condition [*F*(1,81) = 78.98, *p* < 0.001, $\eta_{p}^{2}$ = 0.49] and test time and income [*F*(1,81) = 7.12, *p* = 0.009, $\eta_{p}^{2}$ = 0.08]. However, a significant three-way interaction among time, condition, and income qualified the aforementioned results [*F*(1,81) = 8.155, *p* = 0.005, $\eta_{p}^{2}$ = 0.09] by indicating that condition impacted SVA differently for high- and low-income participants. See **Figure 1**. According to Bonferroni-corrected simple effects tests, for the control group there was no improvement from SVA pretest scores to immediate posttest scores for low-income participants or high-income participants, *F*(1,20) = 2.719, *p* = 0.115,$\eta_{p}^{2}$ = 0.120 and *F*(1,17) = 1.338, *p* = 0.263, $\eta_{p}^{2}$ = 0.073 respectively. However, for the experimental condition, the SVA scores of both the low- and high-income participants improved from pretest to immediate posttest, *F*(1,21) = 102.037, *p* < 0.001, $\eta_{p}^{2}$ = 0.829 and *F*(1,23) = 55.051 *p* < 0.001, $\eta_{p}^{2}$ = 0.705, respectively. Bonferroni-corrected simple effects tests also compared the scores of the high- and low-income participants in the experimental condition on the pretest as well as the immediate posttest. The high-income participants in the experimental condition had higher pretest scores than the low-income participants, F(1,44) = 12.357, *p* = 0.001, $\eta_{p}^{2}$ = 0.219. But, there was no significant difference between the high-income participants immediate posttest scores and the low-income participants’ immediate posttest scores, F(1,44) = 2.812, *p* = 0.101, $\eta_{p}^{2}$ = 0.060. See **Table 1**.

**Table 1:** Mean selective visual attention pretest, immediate posttest, and delayed posttest scores for high- and low-income participants in the control and experimental conditions.

Condition	Pretest	Immediate posttest	Delayed posttest

	*M* (SD)	*M* (SD)	*M* (SD)
**Experimental**			
High-income participants	529.500 (77.009)	589.833 (57.445)	592.023 (44.021)
Low-income participants	452.227 (71.594)	558.909 (67.553)	555.624 (64.659)
**Control**			
High-income participants	513.000 (57.687)	522.722 (53.087)	530.364 (54.387)
Low-income participants	420.048 (33.026)	428.191 (37.782)	425.102 (41.023)

### GOAL TWO

To determine if the SVA benefits caused by acute aerobic exercise lasted 45-min, a 2 (SVA: immediate post test, delayed post test) × 2 (income-level: low-income, high-income) mixed design ANOVA was run for those participants in the experimental condition. There were no significant main effects of income-level [*F*(1,44) = 2.104, *p* = 0.154, $\eta_{p}^{2}$ = 0.046] or test time [*F*(1,44) = 0.944, *p* = 0.337, $\eta_{p}^{2}$ = 0.021], indicating the SVA scores immediately following the exercise session were the same as the SVA scores 45-min later for all participants in the experimental condition. See **Figure 1**.

### GOAL THREE

**FIGURE 2:**
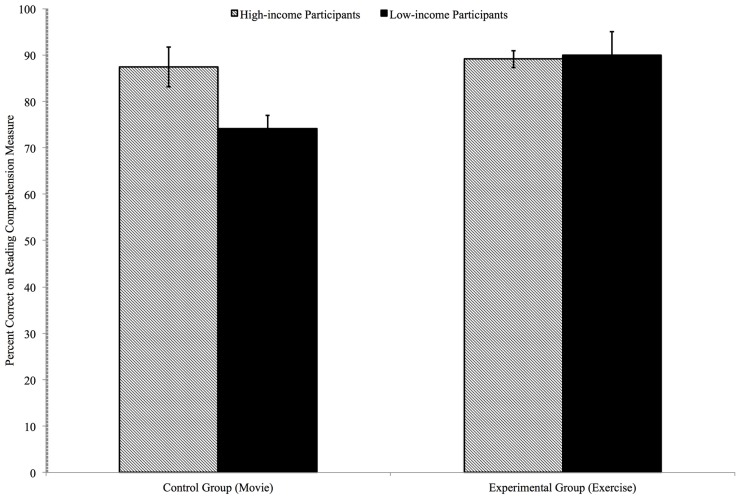
**Reading comprehension as a function of exercise and income level**.

To test if there were differences in reading comprehension scores of the low- and high-income adolescents in the experimental and control conditions, a 2 (condition: experimental, control) × 2 (income-level: low-income, high-income) ANOVA was run with reading comprehension percent correct as the dependent variable. There was a significant main effect of condition [*F*(1,84) = 6.973, *p* = 0.010, $\eta_{p}^{2}$ = 0.079], indicating that the reading comprehension scores of participants in the experimental condition (*M* = 0.896, SD = 0.178) were higher than participants in the control condition (*M* = 0.797, SD = 0.154). There was no significant main effect of income level [*F*(1,84) = 3.641, *p* = 0.060], indicating that the high-and low-income participants had comparable reading comprehension scores (*M* = 0.884, SD = 0.188 and *M* = 0.817, SD = 0.153, respectively). Importantly, there was a significant interaction of condition and income level [*F*(1,84) = 4.595, *p* = 0.035, $\eta_{p}^{2}$ = 0.054]. See **Figure 2** for a graph depicting the reading comprehension scores as a function of condition and income. Bonferroni corrected simple effects tests indicated that there was a difference in the reading comprehension scores of the low- and high-income participants in the control condition, with the low-income participants’ reading comprehension scores (*M* = 0.731, SD = 0.100) being significantly lower than the high-income participants’ reading comprehension scores (*M* = 0.874, SD = 0.172), *F*(1,38) = 10.444, *p* = 0.003. However, that difference did not exist in the experimental condition, where the low-income participants’ scores (*M* = 0.900, SD = 0.152) were not statistically different from the high-income participants’ scores (*M* = 0.891, SD = 0.202), *F*(1,45) = 0.025, *p* = 0.876. Bonferroni corrected simple effects tests also indicated that the reading comprehension scores of the high-income participants in the experimental group and control group were not significantly different from one another [*F*(1,41) = 0.088, *p* = 0.768]. However, the reading comprehension scores of the low-income participants in the experimental and control group were significantly different from one another; the low-income participants in the experimental group had significantly higher reading comprehension scores than the low-income participants in the control group, [*F*(1,42) = 18.381, *p* < 0.001, $\eta_{p}^{2}$ = 0.490].

Correlation analyses were also run to determine the relationship between reading comprehension scores and immediate posttest scores for different participant groups. A set of Fisher-X transformation tests determined if the correlations for different participant groups were significantly different from one another. The correlation between reading comprehensions scores and immediate posttest scores was statistically significant for low-income participants in both the experimental and control condition (*p* < 0.005 in both cases). This was not the case for high-income participants; the correlation between reading comprehension and immediate posttest scores was not statistically significant for high-income participants in the experimental or control condition (*p* > 0.05 in both cases). See **Table 2** for correlation and Fisher’s Z-transformation statistics.

**Table 2:** Correlations between reading comprehension scores and SVA immediate posttest scores for various participant groupings.

Participant type	*r (p*-value)	*R*^2^		*Z (p*-value)
Full sample	**0.540 (0.000)**	0.292		
Experimental	**0.402 (0.006)**	0.162	>	-1.13 (0.253)
Control	**0.592 (0.000)**	0.350		
Low-income	**0.799 (0.000)**	0.638	>	**3.83 (0.000)**
High-income	0.230 (0.143)	0.053		
Low-income experimental	**0.702 (0.000)**	0.493	>	**2.07 (0.039)**
High-income experimental	0.213 (0.318)	0.045		
Low-income control	**0.700 (0.007)**	0.490	>	**1.65 (0.049)**
High-income control	0.283 (0.255)	0.080		

*Bold values indicates statistical significance.*

### ANCILLARY ANALYSES

Ancillary analyses were run to investigate the role life stressors might play in the aforementioned results. According to a 2 (income-level: low-income, high-income) × 2 (condition: experimental, control) ANOVA, there was a main effect of income-level, with low-income participants reporting higher life stress scores (*M* = 36.957, SD = 4.344) than high-income participants (*M* = 21.722, SD = 4.249), *F*(1,84) = 25.496, *p* = < 0.001, $\eta_{p}^{2}$ = 0.239. There was no main effect of condition [*F*(1,84) = 0.452, *p* = 0.504, $\eta_{p}^{2}$ = 0.006] and no interaction of income-level by condition [*F*(1,84) = 0.007, *p* = 0.934, $\eta_{p}^{2}$ = 0.001]. Simple linear regressions were also run to determine if life stress scores explained a significant portion of variance in SVA improvement. SVA improvement scores were calculated by subtracting each participants pretest score from their immediate posttest score. For participants in the experimental group, life stress score explained a significant portion of variance in SVA improvement score, *R*^2^ = 0.103, *B* = 1.082 (SE = 0.480), *F*(1,44) = 5.077, *p* = 0.029. For participants in the control group, life stress score did not explain a significant portion of variance in SVA improvement score, *R*^2^ = 0.082, *B* = 0.501 (SE = 0.275), *F*(1,37) = 3.311, *p* = 0.077.

## DISCUSSION

The first goal of the current study was to determine if an acute session of aerobic exercise improved the SVA of low- and high-income adolescents. The results suggest that it did. More specifically, watching a 12-min movie did not increase the SVA of the low- or high-income adolescents in the control group. However, exercising at an aerobic level for 12-min did improve the SVA of the low- and high-income adolescents in the experimental group. The SVA improvement among the low-income adolescents was particularly large, evidenced by the effect size of 0.829. An effect size of this magnitude suggests that the SVA improvement experienced among the low-income adolescents has veritable practical significance. In fact, the low-income adolescents improved so much that the pre-existing income gap in SVA was eliminated. The low-income adolescents began with lower SVA pretest scores compared to the high-income adolescents. However, after just 12-min of acute aerobic exercise, the low-income adolescents’ SVA scores no longer fell below the high-income adolescents’ SVA scores. See **Figure 1**.

There are a few aspects to this result that are worthy of discussion. The finding that the low-income adolescents’ SVA pretest scores were lower than the high-income adolescents’ SVA pretest scores was expected, as a growing body of literature has documented that low-income children exhibit attention deficits compared to high-income children (e.g., Mezzacappa, 2004; Farah et al., 2005, 2006; Noble et al., 2005, 2007). However, this study is the first to our knowledge to document this income gap in SVA among adolescents, which extends the current literature and highlights that income related attention deficits do not naturally resolve themselves over the course of development. The finding that the improvement among low-income adolescents was slightly larger than the improvement among high-income adolescents also parallels previous work. A similar pattern in SVA improvement was found after low- and high-income children engaged in 12-min of aerobic exercise, with low-income children showing even greater improvement than high-income children (Tine and Butler, 2012). Yet, in the work with children the improvement was not large enough to close the income gap in SVA (Tine and Butler, 2012). In the current study, the improvement among low-income adolescents *was* substantial enough to close the income gap in SVA, which is a novel finding and makes acute aerobic exercise a particularly compelling intervention for low-income adolescents.

Why would an acute session of aerobic exercise impact low-income adolescents more than high-income adolescents? One possibility is that low-income adolescents improved more because they had more room to improve. Another possibility is that low-income adolescents experience more chronic stress than high-income adolescents and the amount of chronic stress experienced affects the physiological response to acute aerobic exercise. As previously mentioned, this hypothesis stems from findings that chronic stress deregulates the same physiological systems that acute aerobic exercise activates (Evans and English, 2002; Kunz-Ebrecht et al., 2004; Viru and Viru, 2004; Cohen et al., 2006; Ferris et al., 2007; Miller et al., 2007; Chen et al., 2010; Griffin et al., 2011). Physiological measures were not collected in this study, but low- and high-income adolescents did report experiencing different amounts of chronic stress, with low-income adolescents reporting more chronic stress than high-income adolescents. This finding aligns with extant literature (e.g., Evans and English, 2002; Almeida et al., 2005). While the finding may not be surprising, it is discouraging because preventative efforts to reduce the chronic stress related poverty are quite complicated to implement. Yet, it is important to highlight that for participants in the experimental condition the amount of chronic stress experienced predicted SVA improvement. Adolescents who experienced more chronic stress improved more than those who experienced less chronic stress. Total chronic stress did not predict SVA improvement for those in the control condition, which makes sense when considering that neither the high- nor low-income adolescents in the control condition experienced SVA improvement from watching the movie. The finding that the group that experiences the most chronic stress can experience such large cognitive and educational benefits from acute aerobic exercise is encouraging and highlights the value of remediation efforts.

The second goal of the current study was to determine if the SVA benefit caused by an acute session of aerobic exercise lasted beyond the exercise session itself. Indeed, the acute session of aerobic exercise improved the SVA of low- and high-income adolescents for 45-min. The sustained benefit of acute aerobic exercise underscores how useful it can be as an intervention. Forty-five minutes is a common length for many high school and college classes. Thus, it could be hypothesized that engaging in 12-min of acute aerobic exercise just prior to a class could result in improved SVA throughout the duration of the class.

Finally, the third goal of the current study was to determine if the reading comprehension scores of adolescents who exercised were higher than those who did not exercise. The mean reading comprehension score of the *low-income* adolescents who engaged in 12-min of acute aerobic exercise was higher than the mean reading comprehension scores of the low-income adolescents who watched a 12-min movie. The effect size (0.490) suggests that the difference between the two groups was moderate in magnitude.

Unexpectedly, the mean reading comprehension score of the high-income adolescents who exercised for 12-min (90%) was not statistically higher than mean reading comprehension score of the high-income adolescents who watched a movie for 12-min (87%).

One possible explanation for this finding is that the high-income participants experienced a ceiling effect on the reading comprehension measure and, as a result, any potential benefits from the exercise were unable to be recorded. Another possibility is that high-income adolescents utilize different cognitive processes than low-income adolescents when completing reading comprehension tasks. For example, perhaps low-income adolescents utilize SVA for reading comprehension, which is affected by acute aerobic exercise, but high-income adolescents utilize other cognitive processes, which are not affected after acute aerobic exercise. The data provides some support for this possibility. The relationship between reading comprehension scores and SVA immediate posttest scores was significant for low-income adolescents, suggesting they utilize SVA when engaged in reading comprehension. However, the relationship was not significant for high-income adolescents, suggesting they may not utilize SVA when engaged in reading comprehension. It is certainly possible that high-income adolescents utilize other cognitive processes, as many cognitive processes are involved in reading comprehension. Even the most basic of the empirically based models of reading comprehension (e.g., simple view of reading, Gough and Tunmer, 1986) attest that reading comprehension utilizes numerous cognitive processes, including but not limited to SVA, decoding, metacognition, cipher and lexical knowledge, phoneme awareness, and reading fluency (Gough and Tunmer, 1986; Juel et al., 1986; Kirby et al., 2003; Johnston and Kirby, 2006). Future work should attempt to more fully determine if different individuals of different income-levels utilize these cognitive processes to different degrees when engaged in reading comprehension and other academic tasks.

As expected, an income gap in reading comprehension was present within the control group; the low-income adolescents obtained a mean score of 73%, which was significantly lower than high-income adolescents’ mean score of 87%. This income gap in reading comprehension is, unfortunately, a common phenomenon (e.g., Reardon, 2013). Yet, there was no income gap in reading comprehension in the experimental group. The low-income adolescents who engaged in a 12-min session of aerobic exercise had reading comprehension scores comparable to their high-income counterparts, with both groups obtaining mean reading comprehension scores of approximately 90%. The finding that there was no income gap in reading comprehension after just 12 min of aerobic exercise is especially meaningful, because reading comprehension is considered by many to be one of the most essential academic abilities, as it is a skill associated with achievement in every academic domain (RAND Reading Study Group, 2002).

A few limitations about the study should be noted. The adolescents in the sample all attended a highly selective liberal arts college and may not be representative of all adolescents. The low-income adolescents did show typical low-income adolescent patterns of achievement; the SVA pretest scores of low-income adolescents were lower than those of the high-income adolescents and the reading comprehension scores of the low-income adolescents in the control group were lower than those of the high-income adolescents in the control group. Still, caution should be taken before assuming that the results drawn from this sample generalize to all adolescent students. Also, it would have been ideal if a reading comprehension pretest had been administered. Participants were randomly assigned to the experimental and control conditions so it can be assumed that the reading comprehension abilities of the two groups were roughly equivalent at the outset and, therefore, observed difference between the conditions can be linked to the intervention and not a characteristic of the individuals in the group (Shadish et al., 2002; Hinkelmann and Kempthorne, 2008; Pearl, 2009). However, random assignment does not guarantee that the two conditions were matched or equivalent in baseline reading comprehension, only that any differences that might exist were due to chance. As a result, the findings cannot be interpreted to mean that acute aerobic exercise improved the reading comprehension of low-income adolescents, only that the low-income adolescents who engaged in acute aerobic exercise had better reading comprehension after they exercised than the low-income adolescents who did not exercise.

The current study has clear educational implications. In fact, the experimental condition was methodologically designed so that could be easily replicated in schools. For example, the only equipment needed that schools may not already have in inventory is a set of reasonably priced heart rate monitors. Also, a 12-min exercise session is brief enough to be realistically integrated into a typical school day. Physical education (PE) classes could intentionally be scheduled before academic classes. PE classes could also be required to include acute aerobic exercise, thereby allowing students to reap cognitive and academic benefits as well as the intended physical benefits. Unfortunately, instead of capitalizing on PE, there is a current decrease in the number of high schools offering PE and PE is rarely a required college requirement (National Association for Sport and Physical Education, 2010). Further, PE classes are being cut most often from schools in lower-income communities (National Association for Sport and Physical Education, 2010).

In summary, an acute session of aerobic exercise improved the SVA of both low-and high-income adolescents. The improvement seen in the low-income adolescents was so large that their SVA abilities matched those of the high-income adolescents after just 12-min of aerobic exercise. Further, the benefit to SVA lasted for 45-min for both low- and high-income adolescents. Moreover, the mean reading comprehension score of low-income adolescents who engaged in an acute session of aerobic exercise was higher than that of low-income adolescents who did not engage in any exercise. In fact, the mean reading comprehension score of low-income adolescents who engaged in an acute session of aerobic exercise was as high as their high-income counterparts’ mean reading comprehension score. The field of education is in need of research-based interventions that are low in cost and easy to implement in low-income communities (Institute of Educational Sciences, 2014). Based on the results of this study, we encourage the implementation of an acute session of aerobic exercise as such an intervention.

## Conflict of Interest Statement

The author declares that the research was conducted in the absence of any commercial or financial relationships that could be construed as a potential conflict of interest.
